# Optimization Design and Nonlinear Bending of Bio-Inspired Helicoidal Composite Laminated Plates

**DOI:** 10.3390/ma16134550

**Published:** 2023-06-23

**Authors:** Taoye Lu, Hui-Shen Shen, Hai Wang, Xiuhua Chen, Miaolin Feng

**Affiliations:** 1 Zhe Jiang Key Laboratory of General Aviation Operation Technology, General Aviation Institute of Zhejiang Jiande, Jiande 311600, China; lutaoye@sjtu.edu.cn; 2 School of Aeronautics and Astronautics, Shanghai Jiao Tong University, Shanghai 200240, China; hsshen@sjtu.edu.cn (H.-S.S.); wanghai601@sjtu.edu.cn (H.W.); 3 School of Ocean and Civil Engineering, Shanghai Jiao Tong University, Shanghai 200240, China; mlfeng@sjtu.edu.cn

**Keywords:** Bouligand structure, simulation-based optimization, large deflection, finite element model

## Abstract

Inspired by the bionic Bouligand structure, helicoidal carbon fiber-reinforced polymer composite (CFRPC) laminates have been proven to own outstanding out-of-plane mechanical properties. This work aims to further explore the excellent bending characteristics of helicoidal CFRPC laminated plates and find out the optimal helicoidal layup patterns. The optimization design of laminated plates stacked with single-form and combination-form helicoidal layup sequences are carried out by using the finite element method (FEM) and adaptive simulated annealing (ASA) optimization algorithm on the Isight platform. Then, the nonlinear bending responses of optimal helicoidal CFRPC laminated plates are investigated via the FEM for the first time. The helicoidal CFRPC laminated plates under three different types of boundary conditions subjected to transverse uniformly distributed load are considered. The numerical results reveal that the combination-form helicoidal layup sequences can decrease the dimensionless bending deflection of laminated plates by more than 5% compared with the quasi-isotropic plate and enhance the out-of-plane bending characteristics of CFRPC laminated plates effectively. The boundary conditions can significantly influence the nonlinear bending responses of helicoidal CFRPC laminated plates.

## 1. Introduction

The Bouligand structure [1], which can be commonly found in various crustacean exoskeletons and insect cuticles [2,3,4], is the natural helicoidal structure and contributes to high transverse load resistance. Carbon fiber-reinforced polymer composite (CFRPC) laminated plates, stacked by designed unidirectional plies with different layup angles, are widely used in aerospace and marine engineering. Motivated by the natural Bouligand structure, the CFRPC laminated plates are designed to have bio-inspired helicoidal layups, enhancing out-of-plane mechanical performance in recent years.

Many researchers have examined the outstanding out-of-plane mechanical properties of helicoidal composite laminated plates. Apichattrabrut and Ravi-Chandar [5] concluded that the smaller angle change between adjacent plies will lead to lower interlaminar shear stress, which results in better damage tolerance of helicoidal CFRPC laminates than unidirectional and cross-ply laminates under tension, bending and impact load. Cheng et al. [6,7,8] first explained the mechanical responses of the exoskeleton of selected crustaceans with helicoidal structures. They showed the improvement in the bending and shear properties of helicoidal composite laminates via experiments and theoretical studies. Liu et al. [9,10] pointed out that, for the thicker helicoidal plates, a smaller rotation angle may promote the transverse propagation of cracks and not always result in a higher peak load under transverse load testing [9]. Jiang et al. [11] firstly introduced three nonlinear helicoidal layup arrangements—recursive, exponential and semicircular—into composite laminated plates and proved the enhancement of the impact resistance of recursive and exponential helicoidal plates with increasing rotation angles. Mencattelli and Pinho [12,13] demonstrated the feasibility of using Bouligand structures to improve the out-of-plane mechanical properties of thin-ply and ultra-thin-ply CFRPCs via experimental and numerical investigations. Mohamed et al. [14] compared the bending properties of CFRPC laminated plates stacked with conventional layups, one kind of linear layup and Fibonacci helicoidal layups. In their studies, the influence of the lamination scheme of the plates was discussed numerically.

The effect of helicoidal layups on the enhancement of the out-of-plane properties of laminated plates has been widely proved. However, the comparison works in the published literatures were only conducted on predefined helicoidal patterns. Thus, the helicoidal CFRPC laminated plates with various rotation angles need to be investigated more comprehensively. In order to obtain helicoidal laminated plates with better bending behaviors, optimization work is introduced in this paper. The common optimization objective for the bending problem is minimizing the deflection. Topology optimization was applied by Pedersen [15] to choose the optimal laminated plates subjected to prestresses. Based on a genetic algorithm used together with the finite element method (FEM), Almeida and Awruch [16] obtained optimal composite laminated structures with minimum weight and deflection under a transverse load. Monte et al. [17] used the direct search method to optimize the layup sequences of composite plates. However, to the knowledge of the authors, the layup optimization on bending behaviors of helicoidal CFRPC laminated plates is still lacking. In this paper, the optimization process is performed using the global optimization algorithm, adaptive simulated annealing (ASA) optimization algorithm [18], and the optimization objective of minimizing the bending deflection of helicoidal CFRPC laminated plates is considered.

Additionally, in actual aerospace application, many static problems of composite laminated plates cannot be adequately solved via the classical thin-plate theory only. The geometrically nonlinear responses and transverse shear effect in the large-deflection region of laminated plates have been considered in many studies [19,20,21,22,23,24,25]. Based on the first-order shear deformation theory (FSDT), Reddy [26] systematically investigated the nonlinear bending of composite laminated plates. Malekzadeh and Setoodeh [27] successfully carried out the prediction of large deformation on moderately thick laminated plates. For the more accurate shear stress distribution, the higher-order shear deformation theory (HSDT) is developed. Based on Reddy’s third-order shear deformation theory (TSDT) [28], a large amount of research on nonlinear bending problems of laminated plates has been reported [29,30,31,32,33]. At the same time, various non-polynomial HSDTs [34,35,36] have been established to analyze the nonlinear responses of laminated plates in order to establish high computational efficiency. The FEM is frequently used to solve the differential equations and deal with laminated plates under different boundary conditions efficiently [37,38,39]. Nowadays, the FEM has been widely used in the analysis of various mechanical problems for composite structures [40,41,42,43]. However, the nonlinear bending responses of helicoidal CFRPC laminated plates remain unclear, and this also motivates the present study.

In the present work, the out-of-plane bending responses of bio-inspired helicoidal CFRPC laminated plates are examined. Unlike the previous research, the layup optimizations of single-form and combination-form helicoidal patters are conducted, and the nonlinear bending responses of optimal helicoidal CFRPC laminated plates are investigated for the first time. The optimization objective of minimizing the bending deflection for higher out-of-plane bending stiffness under three different boundary conditions is carried out using the ASA algorithm on Isight v5.9-5 software. The calculations of the dimensionless bending deflection in linear analysis for optimization and the central bending deflection and moment for nonlinear bending analysis are carried out by using the FEM with ABAQUS. In this paper, 4 kinds of single-form and 12 types of combination-form linear (L) and nonlinear—i.e., Recursive (R), Fibonacci (F) and Exponential (E)—helicoidal layup configurations are designed. All the helicoidal laminated plates are compared with the conventional quasi-isotropic one, and the optimal helicoidal CFRPC laminated plates are selected for the following nonlinear bending analysis.

## 2. Modelling of Helicoidal CFRPC Laminated Plates

### 2.1. Layup Configuration

**Figure 1:**
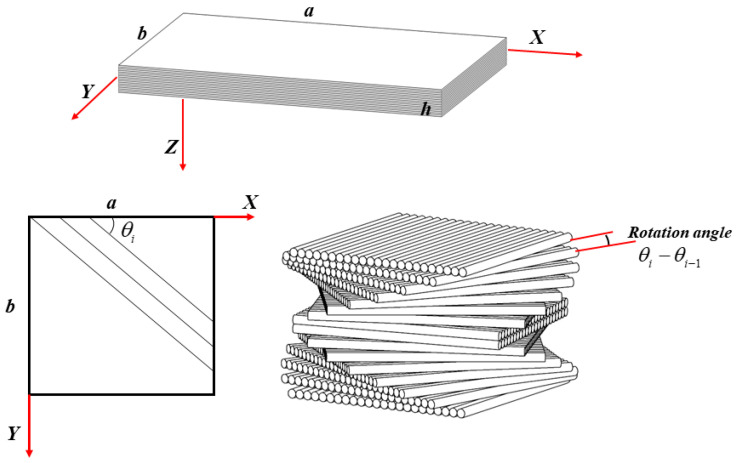
Coordinate system of a helicoidal CFRPC laminated plate.

In this paper, CFRPC laminated plates stacked with two types of single-form and combination-form helicoidal layup configurations are considered. As shown in Figure 1, the helicoidal CFRPC laminated plates contain 16 plies; $\theta_{i}$ refers to the angle between the unidirectional carbon fiber and *X*-axis, and the rotation angle $\theta_{i}-\theta_{i-1}$ refers to the angle difference between adjacent plies. As mentioned before, one linear (L) rotation angle and three nonlinear rotation angles patterns, i.e., recursive (R), Fibonacci (F) and exponential (E), are considered in the present study. The L-helicoidal pattern is the most commonly used in previous studies. Apart from that, some nonlinear helicoidal patterns have been reported recently for the reason that in natural biological helicoidal structures, the rotation angles are always non-equal. Thus, linear and nonlinear helicoidal layup patterns, i.e., the R and E helicoidal patterns proposed by Jiang et al. [11] and the F helicoidal pattern designed by Wang et al. [44], which have been proven to provide outstanding out-of-plane mechanical properties for laminates, are considered in this paper. Thereafter, four kinds of single-form helicoidal layup configurations that rotate according to one of the L, R, F or E helicoidal patterns, and twelve kinds of combination-form helicoidal layup configurations that rotate according to two of the L, R, F or E helicoidal patterns, are designed. The expressions of these helicoidal layup configurations can be expressed as Table 1:

For the L-helicoidal layup configuration, the rotation angle $\theta_{i}-\theta_{i-1}$ is constant and equal to α. For the R-helicoidal layup configuration, $\theta_{i}-\theta_{i-1}$ are in an arithmetic sequence with the tolerance of β. For the F-helicoidal layup configuration, the angle $\theta_{i}$ follows a Fibonacci sequence starting with 0 and γ. For the E-helicoidal layup configuration, the angle $\theta_{i}$ changes exponentially with the base of ξ.

### 2.2. Design Optimization

**Figure 2:**
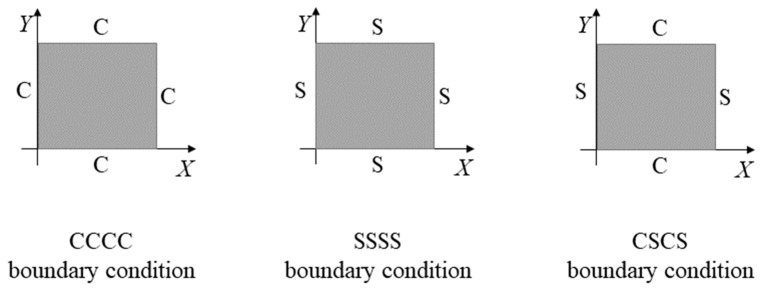
Plates with CCCC, SSSS and CSCS boundary conditions.

In this paper, the bending responses of square bio-inspired helicoidal CFRPC laminated plates subjected to transverse uniformly distributed load are discussed. As reported by Mohamed et al. [14], the boundary conditions (BCs) have significant effects on the bending responses of helicoidal CFRPC laminated plates. In the current study, three types of BCs, namely CCCC, SSSS and CSCS, are considered, as shown in Figure 2. The letter “C” represents the clamped BC and “S” means the simply supported BC.

In the process of optimizing the bending responses of CFRPC laminated plates, the optimal helicoidal layup configurations are searched for to achieve a higher out-of-plane stiffness for the CFRPC laminated plates. The layup optimization problem of the helicoidal laminated plates is non-differentiability, thus, the global optimization algorithm ASA [18] is applied here. Based on a probabilistic function, each new optimal result during the optimization process is probabilistically accepted, so the inferior results can also be accepted in certain probability. The above strategy prevents the ASA optimization process from falling into local optimization. The optimization problem can be formulated as:

(1) $$\left\{\begin{array}{c} Minimize\;\bar{W}\\ s.t.\;\left\{\begin{array}{c} \theta_{i}<2\pi \\ \begin{array}{l} layup\in [L+R,\;L+F,\;L+E,\;R+L,\;R+F,\;R+E,\;\\ \;\;\;\;\;\;\;\;\;\;\;\;\;\;\;\;F+L,\;F+R,\;F+E,\;E+L,\;E+R,\;E+F] \end{array} \end{array}\right.\; \end{array}\right.$$

in which, the angle $\theta_{i}$ of each ply is constrained to less than 360°. For the single-form helicoidal patterns, the number of positive integers of α, β, γ and ξ are limited, so the ASA optimization is only conducted for the combination-form helicoidal patterns, where two of the positive integers of α, β, γ and ξ are specified as the design variables. $\bar{W}$ is the dimensionless bending deflection [28] and can be expressed as:

(2) $$\bar{W}=10^{2}W_{mid}\frac{E_{22}h^{3}}{a^{4}q_{0}}$$

where $W_{mid}$ is the displacement along the thickness direction of the center point on the middle plane. $E_{22}$ is Young’s modulus normal to the fiber direction. *a* and *h* are the length and thickness of the plate, respectively. *q*_0_ is the intensity of the applied uniformly distributed load:

(3) $$q(x,y)=q_{0}$$

**Figure 3:**
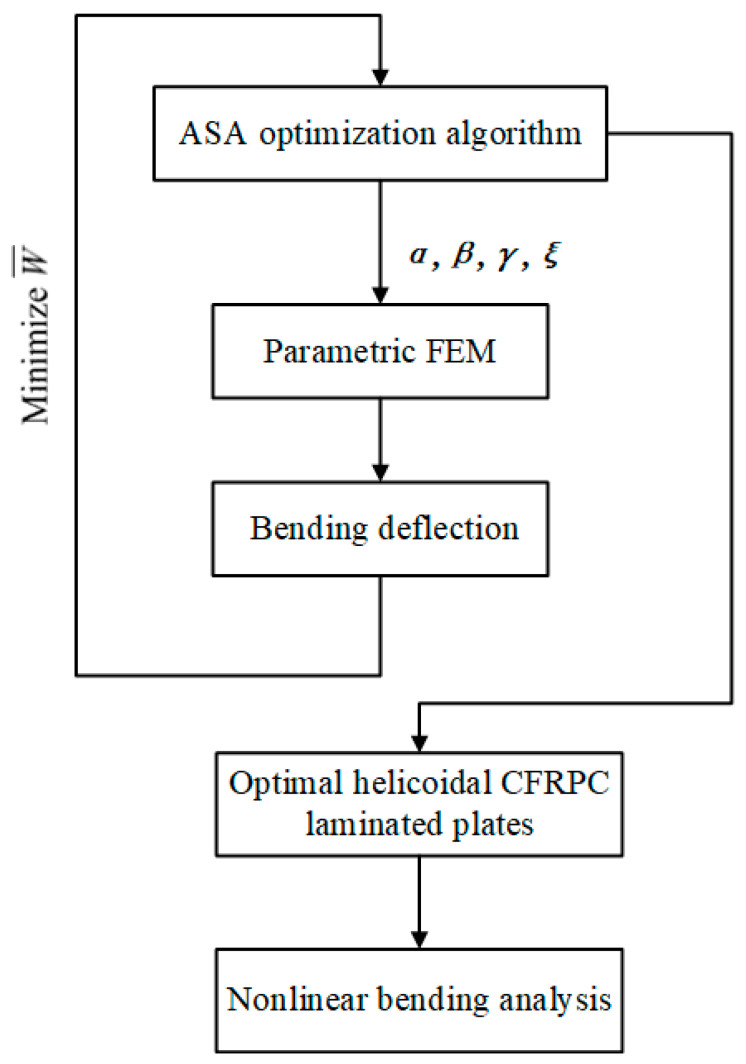
The process of helicoidal layup optimization and nonlinear bending analysis.

The optimization program is operated on the Isight platform. The bending deflection of the laminated plates subjected to transverse uniformly distributed load are obtained in linear analysis using the parametric FEM in ABAQUS. Then, the nonlinear bending analysis is conducted on the optimal helicoidal laminated plates. Figure 3 shows the process of helicoidal layup optimization and nonlinear bending analysis. Isight firstly inputs the new design variables to the FEM. Subsequently, ABAQUS returns the calculated bending deflection to the ASA method. The optimal solution according to the optimization objective of minimizing $\bar{W}$ will be updated, and the new design variables are again input to the FEM for the next optimization cycle. Finally, based on ASA together with the FEM, the nonlinear bending behaviors of the selected optimal helicoidal laminated plates and the QI [0/45/90/−45]_2s_ ones are compared using ABAQUS.

### 2.3. Finite Element Modelling

As shown in Figure 1, consider a square bio-inspired helicoidal CFRPC laminated plate with the geometric size of length *a* = 224 mm, width *b* = 224 mm and thickness *h* = 2.24 mm. The plate is symmetrical and contains 16 plies with an identical thickness of 0.14 mm. A cartesian coordinate system is defined, and its origin point is set at the corner on the middle plane, where *X*, *Y* and *Z* axes are defined along the length, width and thickness directions of the plate.

The bending analyses of CFRPC laminated plates are carried out by using the FEM with ABAQUS. The laminated plate is established using the composite layup editor tool in ABAQUS and is meshed with eight-node brick element C3D8. The material properties of each CFRPC ply are *E*_11_ = 120 GPa, *E*_22_ = *E*_33_ = 11 GPa, *G*_12_ = *G*_13_ = 6.478 GPa, *G*_23_ = 2.518 GPa, *ν*_12_ = *ν*_13_ = 0.33 GPa and *ν*_23_ = 0.35 GPa [39]. The degrees of freedom of the nodes for these three BCs in the FEM are presented in Equation (4).

CCCC:

(4a) $$U_{X}=U_{Y}=U_{Z}=0\left\{\begin{array}{l} \mathrm{at}\;Y=0,b\\ \mathrm{at}\;X=0,a \end{array}\right.$$

SSSS:

(4b) $$U_{X}=U_{Y}=U_{Z}=0\left\{\begin{array}{l} \mathrm{at}\;Y=0,b\;\mathrm{and}\;Z=0\\ \mathrm{at}\;X=0,a\;\mathrm{and}\;Z=0 \end{array}\right.$$

CSCS:

(4c) $$U_{X}=U_{Y}=U_{Z}=0\left\{\begin{array}{l} \mathrm{at}\;Y=0,b\\ \mathrm{at}\;X=0,a\;\mathrm{and}\;Z=0 \end{array}\right.$$

where *U_X_*, *U_Y_* and *U_Z_* represent the translation displacements along the *X*, *Y* and *Z* directions, respectively.

**Figure 4:**
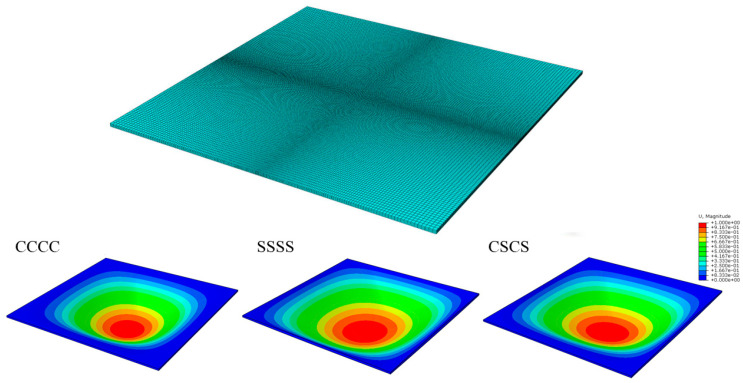
Schematic diagram of FE analysis of the laminated plate and deflection distribution of laminated plates under CCCC, SSSS and CSCS boundary conditions.

The mesh convergence study should be performed before the FE analysis. The dimensionless bending deflection $\bar{W}$ of QI CFRPC laminated plates with different mesh sizes under CCCC, SSSS and CSCS boundary conditions from the FEM are listed in Table 2. Thus, according to the results, in the following bending analysis, all the CFRPC laminated plates are meshed with centrally densified grids and the minimum mesh size is 0.56 × 0.56 × 0.56 mm, as shown in Figure 4.

## 3. Layup Optimization and Nonlinear Bending Analysis

In this section, the layup optimization for minimum bending deflection and the nonlinear bending analysis of helicoidal CFRPC laminated plates with optimal single-form and combination-form helicoidal layups are conducted based on the ASA optimization algorithm (on Isight) together with the FEM (in ABAQUS). Comparison works are carried out with the counterpart QI plate under CCCC, SSSS and CSCS boundary conditions subjected to a transverse uniformly distributed load. The prediction of the bending deflection in the optimization process is carried out in linear analysis. Additionally, in the subsequently nonlinear bending simulation, the geometrical nonlinearity is considered in ABAQUS/Standard with the Nlgeom option turned on.

### 3.1. Validation Studies

***Example 1*:** In this case, the linear central deflection of conventional laminated plates UD ([0]_32_) and QI ([0/45/90/−45]_4s_) together with helicoidal laminated plates LH ([0/24/48/72/96/120/144/168/192/216/240/264/288/312/336/360]_s_) and FH ([0/10/10/20/30/50/80/130/210/340/190/170/360/170/170/340]_s_) under CCCC and SSSS boundary conditions subjected to transverse uniformly distributed load are calculated and compared in Table 2. The plates have *a*/*h* = 100 and *a*/*b* = 1, and the material properties used here are: *E*_11_/*E*_22_ = 25, *E*_22_ = *E*_33_, *G*_12_ = *G*_13_ = 0.5*E_2_*_2_, *G*_23_ = 0.2*E_2_*_2_, *ν*_12_ = *ν*_13_ = *ν*_23_ = 0.25. In the present FE simulation, the boundary conditions are set as mentioned in Section 2.3. As shown in Table 3, good agreements can be observed between the dimensionless bending deflection obtained from the present FEM and those obtained by Mohamed [14].

**Figure 5:**
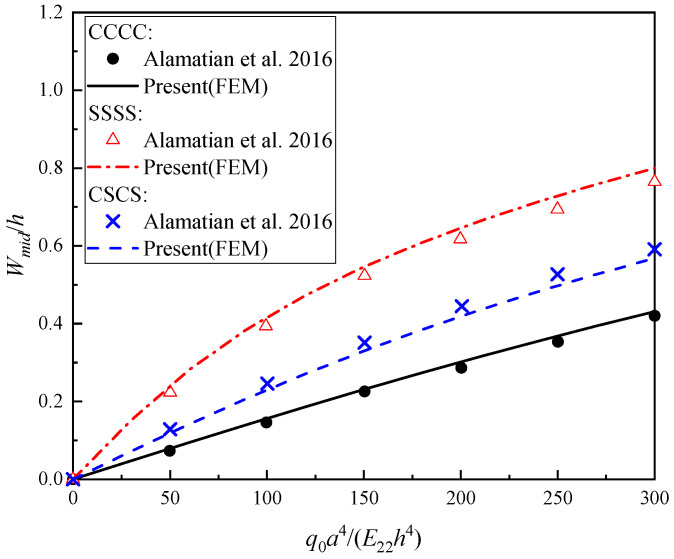
Comparison of the nonlinear bending load–central deflection curves of composite laminated plates under CCCC, SSSS and CSCS boundary conditions subjected to a transverse uniformly distributed load with Alamatian et al. [45].

***Example 2:*** In this example, a 16-ply square [0/45/−45/90]_4_ laminated plate with the geometric size of *a*/*b* = 1 and *a*/*h* = 100 is considered. The CCCC, SSSS and CSCS boundary conditions are set as mentioned in Section 2.3. The material properties [45] are: *E*_11_/*E*_22_ = 25, *E*_22_ = *E*_33_, *G*_12_ = *G*_13_ = 0.5*E*_22_, *G*_23_ = 0.2*E*_22_, *ν*_12_ = *ν*_13_ = *ν*_23_ = 0.25. The nonlinear bending load–central deflection curves of the composite lamianted plates are presented in Figure 5, from which a good agreement can be observed again.

### 3.2. Optimization of Helicoidal Layups for Minimum Bending Deflection

The layup optimization studies for better out-of-plane bending responses of helicoidal CFRPC laminated plates are carried out. The dimensionless bending deflection $\bar{W}$ of symmetrical square helicoidal CFRPC laminated plates stacked with either only one (single-form) or a combination of two (combination-form) of L, R, F and E helicoidal arrangements are compared with the conventional QI, i.e., [0/45/90/−45]_2s_ subjected to atransverse uniformly distributed load.

Table 4, Table 5 and Table 6 show the dimensionless bending deflection $\bar{W}$ of optimal bio-inspired helicoidal laminated plates with a layup sequence of only one of the L, R, F or E helicoidal arrangements (called single-form) under CCCC, SSSS and CSCS boundary conditions. For these single-form helicoidal plates with limited design variables of α, β, γ and ξ, the bending deflection of all four types of helicoidal laminates subjected to a transverse uniformly distributed load are calculated and listed in Table 4, Table 5 and Table 6.

The dimensionless bending deflections $\bar{W}$ for optimal single-form helicoidal laminated plates under the CCCC boundary condition in Table 4 show that the F helicoidal laminated plate with ([0/1/1/2/3/5/8/13]_s_) has the lowest bending deflection, $\bar{W}$ = 0.2933, which is 6.278% lower than that of the QI plate. The bending deflection of the optimal L, R and E helicoidal plates decreased by 6.261%, 5.743% and 4.973%, respectively, compared to the QI plate. It can be observed from the results in Table 4 that the single-form helicoidal laminated plates with lower rotation angles tend to have higher out-of-plane bending stiffness under the CCCC boundary condition subjected to a transverse uniformly distributed load.

For the minimum dimensionless bending deflection $\bar{W}$ of CFRPC laminated plates under the SSSS boundary condition, Table 5 gives the optimal single-form helicoidal layups. Among the four single-form helicoidal laminated plates, the L helicoidal laminated plate with ([0/51/102/153/204/255/306/357]_s_) decreases the bending deflection by 0.365%. However, for the other three single-form helicoidal layups, no improvement in the out-of-plane bending stiffness can be observed, with the bending deflection of these plates being higher than that of the QI plate. It is suggested that the enhancement of the out-of-plane bending stiffness of the helicoidal CFRPC laminated plates stacked with single-form helicoidal arrangements under the SSSS boundary condition subjected to a transverse uniformly distributed load are limited.

Table 6 lists the optimal helicoidal laminated plates stacked with single-form helicoidal layups for the minimum dimensionless bending deflection $\bar{W}$ under the CSCS boundary condition. Similar to the observation for plates under the SSSS boundary condition, only the L-helicoidal laminated plate with ([0/43/86/129/172/215/258/301]_s_) can decrease the bending deflection by 0.113%, and the other three single-form helicoidal plates cannot decrease the bending deflection. It is suggested that the single-form helicoidal arrangements do not contribute significantly to improving the out-of-plane bending stiffness of CFRPC laminated plates under the CSCS boundary condition subjected to a transverse uniformly distributed load.

The optimal combination-form helicoidal CFRPC laminated plates for minimum dimensionless bending deflection $\bar{W}$ under the CCCC, SSSS and CSCS boundary conditions subjected to a uniformly distributed load are listed in Table 7, Table 8 and Table 9. For these CFRPC laminated plates stacked with two of the L, R, F or E helicoidal layup patterns, the ASA optimization algorithm operated on the Isight platform together with ABAQUS is applied.

The optimization results in Table 7 show that all the combination-form helicoidal layup patterns can improve the out-of-plane bending stiffness under the CCCC boundary condition compared to the QI plate. Among them, the optimal (F + L) helicoidal plate of [0/179/179/358/0/1/2/3]_s_ has the lowest dimensionless bending deflection $\bar{W}$ of 0.2931, which is 6.353% lower than that of the QI plate. All the combination-form helicoidal layups can improve the out-of-plane bending stiffness of the CFRPC plate by more than 5% compared to the QI plate. Additionally, under the CCCC boundary condition, the smaller the angle between the adjacent plies, the lower the bending deflection of the laminated plate subjected to a transverse uniformly distributed load. The lower rotation angles of the helicoidal laminated plates enhance the bending stiffness *D*_11_ obviously, which leads to the lower bending deflection.

For combination-form helicoidal CFRPC laminated plates under the SSSS boundary condition, the optimal layup patterns (L + R), (L + F), (R + L), (R + F), (R + E), (F + L) and (F + R) can decrease the dimensionless bending deflection $\bar{W}$ of laminated plates, as shown in Table 8. The lowest $\bar{W}$ of 1.0376, 5.542% lower than the QI plate, is achieved by the (R + F) plate with ([0/50/150/300/0/45/45/90]_s_). Compared to the optimal single-form helicoidal laminated plates under the SSSS boundary condition in Table 5, the improvement in the out-of-plane bending stiffness of plates with combination-form helicoidal layups of (R + L), (R + F) and (R + E) is much more obvious. Compared to the QI ([0/45/90/−45]_2s_) plate with zero *A*_16_ and *A*_26_, the optimal helicoidal laminated plates have the nonzero *A*_16_ and *A*_26_ that offers more shear stiffness than the QI plate.

The results of the layup optimization of combination-form helicoidal patterns for achieving minimum bending deflection for CFRPC laminated plates under the CSCS boundary condition subjected to a transverse uniformly distributed load are presented in Table 9. It can be seen from the optimal helicoidal configurations that (L + R), (L + F), (R + F), (F + L), (F + R) and (F + E) can decrease the $\bar{W}$ of laminated plates compared to the QI plate. The (F + L) plate with ([0/113/113/226/0/47/94/141]_s_) has the lowest $\bar{W}$, 0.4911, which is 5.360% lower than the QI plate. Additionally, compared to the single-form helicoidal laminated plates under the CSCS boundary condition in Table 6, the combination-form (F + L), (F + R) and (F + E) helicoidal layups can enhance the out-of-plane bending stiffness of plates much more effectively.

### 3.3. Nonlinear Bending Behaviors of Optimal Helicoidal CFRPC Laminated Plates

In the previous subsection, the dimensionless bending deflection $\bar{W}$ of the QI and helicoidal laminated plates for linear analysis are calculated. The optimal single-form and combination-form helicoidal CFRPC laminated plates are selected to predict the nonlinear bending responses which consider the geometrical nonlinearity. For the laminated plates under the CCCC boundary condition, the nonlinear bending responses of the optimal single-form F-helicoidal plate of [0/1/1/2/3/5/8/13]_s_ and combination-form (F + L) helicoidal plate of [0/179/179/358/0/1/2/3]_s_ are compared with that of the QI plate. For the laminated plates under the SSSS boundary condition, the optimal single-form L-helicoidal plate of [0/51/102/153/204/255/306/357]_s_ and combination-form (R + F) plate of [0/50/150/300/0/45/45/90]_s_ are selected. Additionally, for the laminated plates under the CSCS boundary condition, the optimal single-form L-helicoidal plate of [0/43/86/129/172/215/258/301]_s_ and combination-form (F + L) plate of [0/113/113/226/0/47/94/141]_s_ are selected. All the plates have the same geometric size, with *a* = 224 mm, *b* = 224 mm and *h* = 2.24 mm, and are subjected to a transverse uniformly distributed load. In the following figures, $W_{mid}$ and $M_{X}$ represent the deflection of the center point on the middle plane and the central bending moment of the plate, respectively.

**Figure 6:**
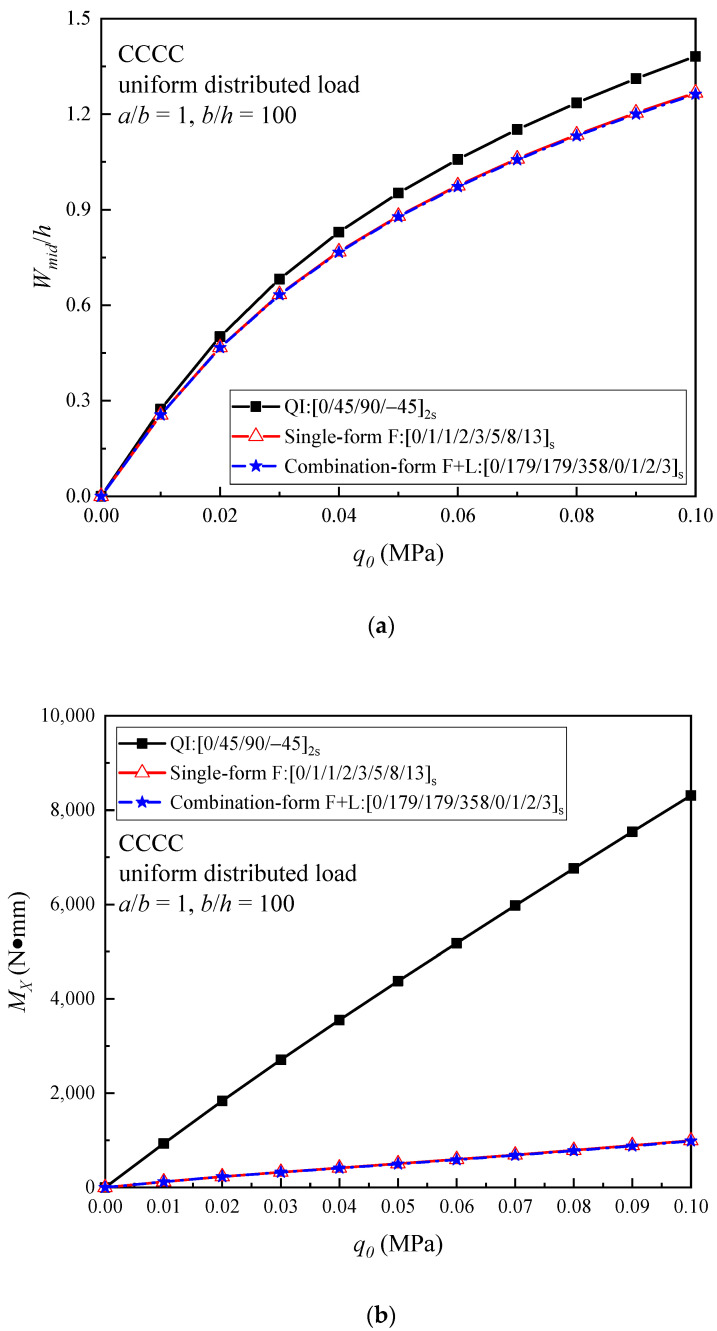
Comparison of (**a**) the load–central deflection curves; and (**b**) the load–bending moment curves of the QI and optimal helicoidal CFRPC laminated plates under the CCCC boundary condition subjected to a uniformly distributed load.

Figure 6 illustrates the nonlinear bending response of the optimal helicoidal plates and QI plate under the CCCC boundary condition. It can be observed that the F and (F + L) helicoidal plates have very close curves which are lower than QI plate for both the load–central deflection and the load–bending moment curves. During the nonlinear bending process, the optimal F and (F + L) helicoidal plates maintain the higher out-of-plane bending stiffness under the CCCC boundary condition subjected to a uniformly distributed load.

**Figure 7:**
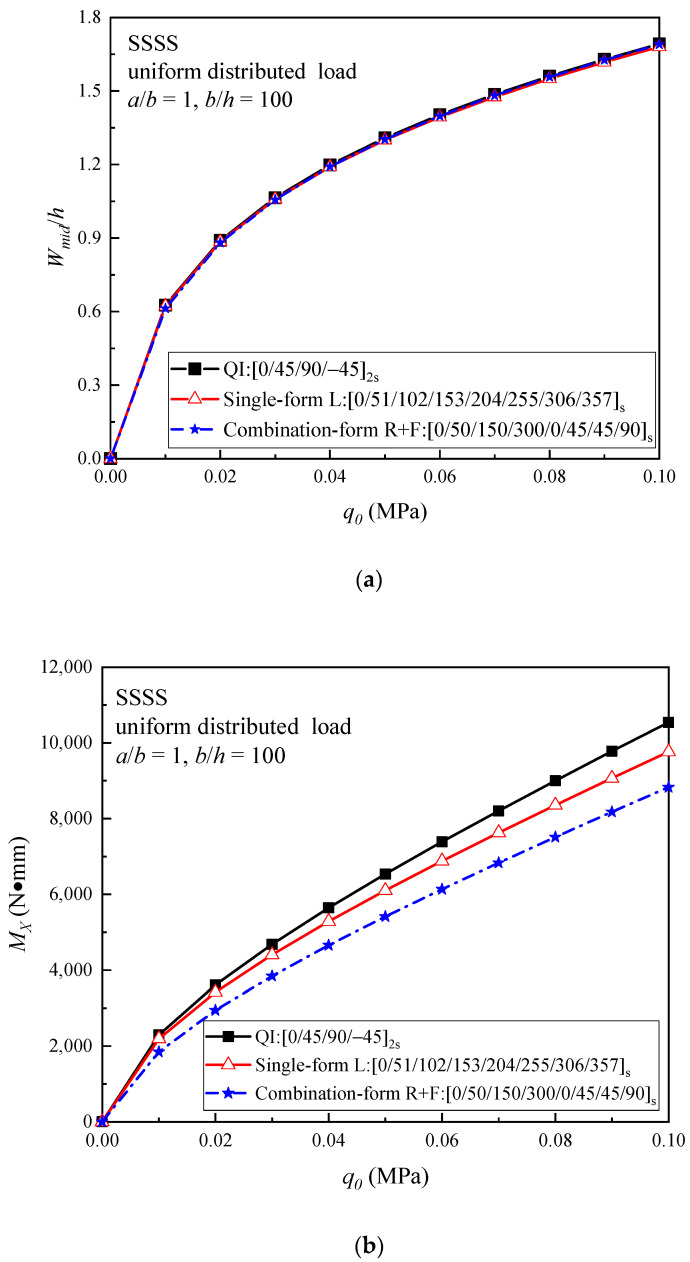
Comparison of (**a**) the load–central deflection curves; and (**b**) the load–bending moment curves of the QI and optimal helicoidal CFRPC laminated plates under the SSSS boundary condition subjected to a uniformly distributed load.

In Figure 7, the relationships between the applied load and the central deflection together with the applied load and the bending moment are presented. The results show that the load–central deflection curves of these three plates are close under the SSSS boundary condition, while the enhancement of the out-of-plane bending properties is not obvious for the optimal helicoidal laminated plates compared with the QI plate during the nonlinear bending process. On the other hand, the load–bending moment curves of the optimal plates are lower than those of the QI plate.

**Figure 8:**
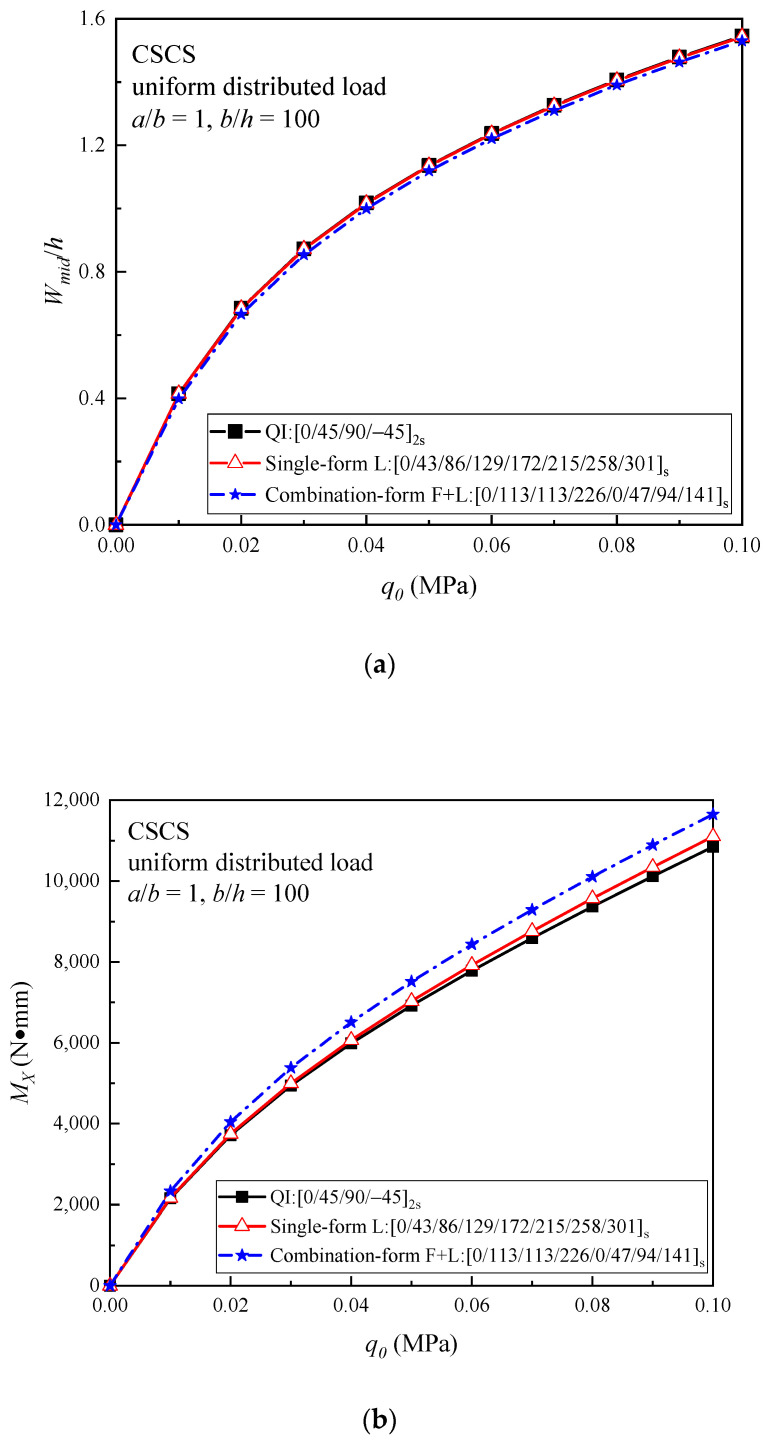
Comparison of (**a**) the load–central deflection curves; and (**b**) the load–bending moment curves of the QI and optimal helicoidal CFRPC laminated plates under the CSCS boundary condition subjected to a uniformly distributed load.

The nonlinear bending responses of the optimal helicoidal laminated plates under the CSCS boundary condition are given in Figure 8. It can be seen from the results that, among the three plates, the (F + L) plate has the lowest load–central deflection curve, but the highest load–bending moment curve. The load–central deflection curves of the optimal L helicoidal plate and QI plate are very close, while the load–bending moment curve of the L plate is slightly higher than that of the QI plate.

## 4. Conclusions

The optimization design and nonlinear bending analysis of bio-inspired helicoidal CFRPC laminated plates under CCCC, SSSS and CSCS boundary conditions subjected to a transverse uniformly distributed load have been presented. The purpose of layup optimization is to find the optimal CFRPC laminated plates stacked with single-form and combination-form bio-inspired helicoidal layups that own the best out-of-plane mechanical properties and which demonstrate the lowest bending deflection when subjected to a transverse uniformly distributed load. The global optimization algorithm ASA based on Isight and associated with the FEM operated in ABAQUS is applied. For the first time, the nonlinear bending responses of the optimal helicoidal CFRPC laminated plates are studied. The notable results are summarized below.

(a) Under the CCCC boundary condition, both the single-form and combination-form helicoidal layups with the lower rotation angles can decrease the bending deflection.
(b) Among the optimal CCCC helicoidal laminated plates, the F helicoidal plate ([0/1/1/2/3/5/8/13]_s_) has the lowest dimensionless bending deflection score, 0.2933, which is 6.278% lower than the QI plate, and the (F + L) helicoidal plate ([0/179/179/358/0/1/2/3]_s_) has the lowest bending deflection of 0.2931, which is 6.353% lower than the QI plate.
(c) Under SSSS and CSCS boundary conditions, the enhancement of the out-of-plane bending properties of the CFRPC laminated plates using single-form helicoidal layup arrangements are limited, while by stacking with the combination-form helicoidal patterns, the linear dimensionless bending deflection of the CFRPC laminated plates can be obviously decreased.
(d) For the optimal SSSS helicoidal laminated plates, the lowest bending deflection of 1.0376, which is 5.542% lower than the QI plate, is achieved by the (R + F) helicoidal plate ([0/50/150/300/0/45/45/90]_s_). Additionally, for the optimal CSCS helicoidal laminated plates, the (F + L) helicoidal plate ([0/113/113/226/0/47/94/141]_s_) has the lowest bending deflection of 0.4911, which is 5.360% lower than the QI plate.
(e) The boundary conditions have significant effects on the nonlinear bending responses of helicoidal laminated plates.

## Figures and Tables

**Table 1:** The single-form and combination-form helicoidal layup configurations.

Single-form helicoidal layup configurations	
L	${\left[0/\alpha /2\alpha /3\alpha /4\alpha /5\alpha /6\alpha /7\alpha \right]}_{s}\Rightarrow \theta_{1}=0,\theta_{i}-\theta_{i-1}=\alpha$
R	${\left[0/\beta /3\beta /6\beta /10\beta /15\beta /21\beta /28\beta \right]}_{s}\Rightarrow \theta_{1}=0,\theta_{i}-\theta_{i-1}=\beta (i-1)$
F	${\left[0/\gamma /\gamma /2\gamma /3\gamma /5\gamma /8\gamma /13\gamma \right]}_{s}\Rightarrow \theta_{1}=0,\theta_{2}=\gamma ,\theta_{i}=\theta_{i-2}+\theta_{i-1}$
E	${\left[\xi /\xi^{2}/\xi^{3}/\xi^{4}\right]}_{2s}\Rightarrow \theta_{i}=\xi^{i}$
Combination-form helicoidal layup configurations	
L + R	${\left[0/\alpha /2\alpha /3\alpha /0/\beta /3\beta /6\beta \right]}_{s}$
L + F	${\left[0/\alpha /2\alpha /3\alpha /0/\gamma /\gamma /2\gamma \right]}_{s}$
L + E	${\left[0/\alpha /2\alpha /3\alpha /\xi /\xi^{2}/\xi^{3}/\xi^{4}\right]}_{s}$
R + L	${\left[0/\beta /3\beta /6\beta /0/\alpha /2\alpha /3\alpha \right]}_{s}$
R + F	${\left[0/\beta /3\beta /6\beta /0/\gamma /\gamma /2\gamma \right]}_{s}$
R + E	${\left[0/\beta /3\beta /6\beta /\xi /\xi^{2}/\xi^{3}/\xi^{4}\right]}_{s}$
F + L	${\left[0/\gamma /\gamma /2\gamma /0/\alpha /2\alpha /3\alpha \right]}_{s}$
F + R	${\left[0/\gamma /\gamma /2\gamma /0/\beta /3\beta /6\beta \right]}_{s}$
F + E	${\left[0/\gamma /\gamma /2\gamma /\xi /\xi^{2}/\xi^{3}/\xi^{4}\right]}_{s}$
E + L	${\left[\xi /\xi^{2}/\xi^{3}/\xi^{4}/0/\alpha /2\alpha /3\alpha \right]}_{s}$
E + R	${\left[\xi /\xi^{2}/\xi^{3}/\xi^{4}/0/\beta /3\beta /6\beta \right]}_{s}$
E + F	${\left[\xi /\xi^{2}/\xi^{3}/\xi^{4}/0/\gamma /\gamma /2\gamma \right]}_{s}$

**Table 2:** Mesh convergence study for the dimensionless bending deflection $\bar{W}$ of the square QI CFRPC laminated plates under CCCC, SSSS and CSCS boundary conditions.

BC	Minimum Mesh Size (mm)	Nodes	$\bar{W}$	Difference
	2.24 × 2.24 × 0.56	51,005	0.3044	-
CCCC	1.12 × 1.12 × 0.56	96,605	0.3109	(0.3109−0.3044)/0.3044 ≈ 2.14%
	0.56 × 0.56 × 0.56	171,125	0.3130	(0.3130−0.3109)/0.3109 ≈ 0.68%
	2.24 × 2.24 × 0.56	51,005	1.0603	-
SSSS	1.12 × 1.12 × 0.56	96,605	1.0891	(1.0891−1.0603)/1.0603 ≈ 2.72%
	0.56 × 0.56 × 0.56	171,125	1.0985	(1.0985−1.0891)/1.0891 ≈ 0.86%
	2.24 × 2.24 × 0.56	51,005	0.5027	-
CSCS	1.12 × 1.12 × 0.56	96,605	0.5149	(0.5149−0.5027)/0.5027 ≈ 2.43%
	0.56 × 0.56 × 0.56	171,125	0.5189	(0.5189−0.5149)/0.5149 ≈ 0.78%

**Table 3:** Comparison of the dimensionless bending deflection $\bar{W}$ of the square composite laminated plates under CCCC and SSSS boundary conditions subjected to a uniformly distributed load.

	CCCC		SSSS	
	Mohamed [14]	Present (FEM)	Mohamed [14]	Present (FEM)
UD	0.1340	0.1387	0.6528	0.6911
QI	0.1535	0.1566	0.5018	0.5301
LH	0.1548	0.1570	0.5111	0.5347
FH	0.1541	0.1579	0.5691	0.5996

**Table 4:** The dimensionless bending deflection $\bar{W}$ of the optimal bio-inspired helicoidal CFRPC laminated plates with a single-form helicoidal layup pattern under the CCCC boundary condition.

Designation	Configuration	$\bar{W}$	Relative Change
QI	[0/45/90/−45]_2s_	0.3130	-
L	[0/1/2/3/4/5/6/7]_s_	0.2934	−6.261% *
R	[0/1/3/6/10/15/21/28]_s_	0.2950	−5.743%
F	[0/1/1/2/3/5/8/13]_s_	0.2933	−6.278%
E	[2/4/8/16]_2s_	0.2974	−4.973%

* Relative change comparing to QI plate = 100% ($\bar{W}$ − 0.3130)/0.3130.

**Table 5:** The dimensionless bending deflection $\bar{W}$ of the optimal bio-inspired helicoidal CFRPC laminated plates with a single-form helicoidal layup pattern under the SSSS boundary condition.

Designation	Configuration	$\bar{W}$	Relative Change
QI	[0/45/90/−45]_2s_	1.0985	-
L	[0/51/102/153/204/255/306/357]_s_	1.0945	−0.365%
R	[0/12/36/72/120/180/252/336]_s_	1.2051	9.706%
F	[0/27/27/54/81/135/216/351]_s_	1.1868	8.041%
E	[3/9/27/81]_2s_	1.3031	18.626%

**Table 6:** The dimensionless bending deflection $\bar{W}$ of the optimal bio-inspired helicoidal CFRPC laminated plates with a single-form helicoidal layup pattern under the CSCS boundary condition.

Designation	Configuration	$\bar{W}$	Relative Change
QI	[0/45/90/−45]_2s_	0.5189	-
L	[0/43/86/129/172/215/258/301]_s_	0.5183	−0.113%
R	[0/12/36/72/120/180/252/336]_s_	0.6498	25.228%
F	[0/27/27/54/81/135/216/351]_s_	0.6769	30.444%
E	[4/16/64/256]_2s_	0.6157	18.645%

**Table 7:** The dimensionless bending deflection $\bar{W}$ of the optimal bio-inspired helicoidal CFRPC laminated plates with a combination-form helicoidal layup pattern under the CCCC boundary condition.

Designation	Configuration	$\bar{W}$	Relative Change
QI	[0/45/90/−45]_2s_	0.3130	-
L + R	[0/1/2/3/0/1/3/6]_s_	0.2932	−6.313%
L + F	[0/1/2/3/0/178/178/356]_s_	0.2932	−6.326%
L + E	[0/1/2/3/2/4/8/16]_s_	0.2934	−6.243%
R + L	[0/1/3/6/0/1/2/3]_s_	0.2935	−6.225%
R + F	[0/1/3/6/0/178/178/356]_s_	0.2935	−6.236%
R + E	[0/1/3/6/2/4/8/16]_s_	0.2938	−6.139%
F + L	[0/179/179/358/0/1/2/3]_s_	0.2931	−6.353%
F + R	[0/179/179/358/0/1/3/6]_s_	0.2931	−6.349%
F + E	[0/179/179/358/2/4/8/16]_s_	0.2933	−6.305%
E + L	[2/4/8/16/0/1/2/3]_s_	0.2970	−5.116%
E + R	[2/4/8/16/0/1/3/6]_s_	0.2970	−5.105%
E + F	[2/4/8/16/0/174/174/348]_s_	0.2968	−5.175%

**Table 8:** The dimensionless bending deflection $\bar{W}$ of the optimal bio-inspired helicoidal CFRPC laminated plates with a combination-form helicoidal layup pattern under the SSSS boundary condition.

Designation	Configuration	$\bar{W}$	Relative Change
QI	[0/45/90/−45]_2s_	1.0985	-
L + R	[0/111/222/333/0/47/141/282]_s_	1.0899	−0.785%
L + F	[0/46/92/138/0/135/135/270]_s_	1.0871	−1.037%
L + E	[0/112/224/336/4/16/64/256]_s_	1.1047	0.570%
R + L	[0/50/150/300/0/36/72/108]_s_	1.0429	−5.059%
R + F	[0/50/150/300/0/45/45/90]_s_	1.0376	−5.542%
R + E	[0/50/150/300/4/16/64/256]_s_	1.0515	−4.276%
F + L	[0/121/121/242/0/32/64/96]_s_	1.0898	−0.791%
F + R	[0/121/121/242/0/44/132/264]_s_	1.0881	−0.944%
F + E	[0/121/121/242/4/16/64/256]_s_	1.0998	0.122%
E + L	[4/16/64/256/0/119/238/357]_s_	1.2649	15.148%
E + R	[3/9/27/81/0/1/3/6]_s_	1.2690	15.527%
E + F	[4/16/64/256/0/136/136/272]_s_	1.2394	12.826%

**Table 9:** The dimensionless bending deflection $\bar{W}$ of the optimal bio-inspired helicoidal CFRPC laminated plates with combination-form helicoidal layup pattern under the CSCS boundary condition.

Designation	Configuration	$\bar{W}$	Relative Change
QI	[0/45/90/−45]_2s_	0.5189	-
L + R	[0/42/84/126/0/43/129/258]_s_	0.5183	−0.128%
L + F	[0/41/82/123/0/103/103/206]_s_	0.5085	−2.006%
L + E	[0/42/84/126/4/16/64/256]_s_	0.5260	1.365%
R + L	[0/43/129/258/0/118/236/354]_s_	0.5251	1.193%
R + F	[0/43/129/258/0/101/101/202]_s_	0.5186	−0.064%
R + E	[0/44/132/264/4/16/64/256]_s_	0.5359	3.275%
F + L	[0/113/113/226/0/47/94/141]_s_	0.4911	−5.360%
F + R	[0/113/113/226/0/43/129/258]_s_	0.4940	−4.812%
F + E	[0/114/114/228/4/16/64/256]_s_	0.4996	−3.730%
E + L	[4/16/64/256/0/119/238/357]_s_	0.5941	14.485%
E + R	[4/16/64/256/0/44/132/264]_s_	0.6024	16.076%
E + F	[4/16/64/256/0/112/112/224]_s_	0.5849	12.720%

## Data Availability

The data presented in this study are available on request from the corresponding author.
